# Supplementary material for: Polyphenols Content, Antioxidant Properties and Allergenic Potency of Organic and Conventional Blue Honeysuckle Berries
Source: Molecules. 2022 Sep 18;27(18):6083. doi: 10.3390/molecules27186083 (PMC9502526; doi:10.3390/molecules27186083)
Supplement: Supplementary file 1 [file molecules-27-06083-s001.zip › molecules-1900402-supplementary.pdf]

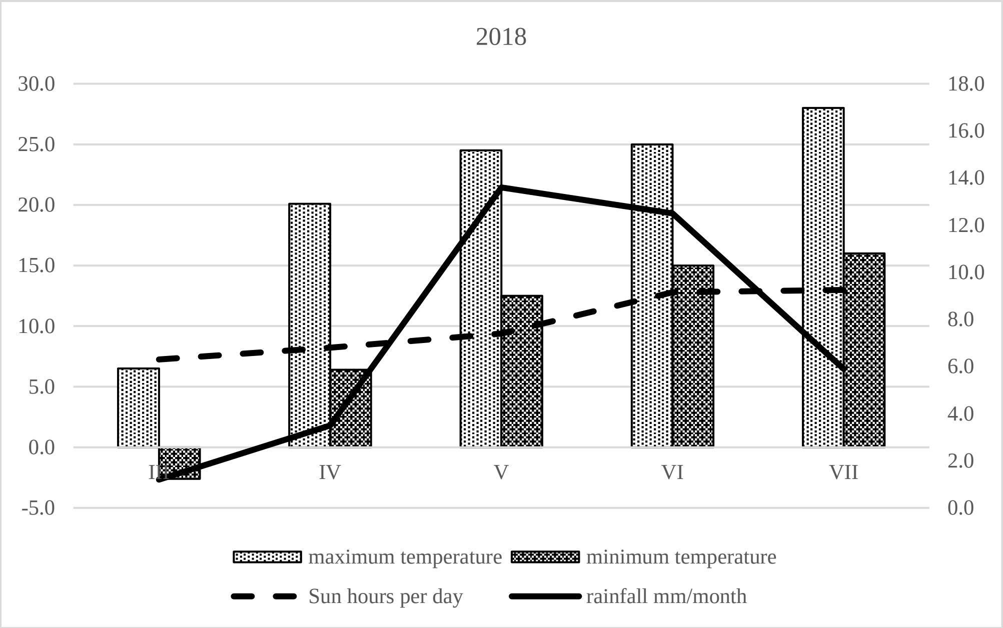

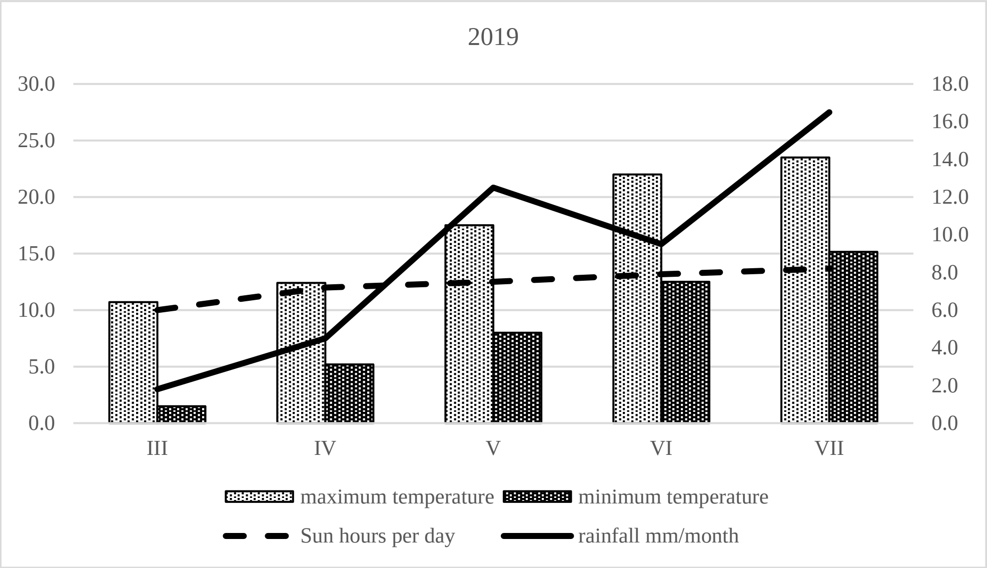

Figure S1. Weather conditions in experimental orchards (organic and conventional) 2018–2019 in time of blue honeysuckle vegetation, flowering and fruits development.

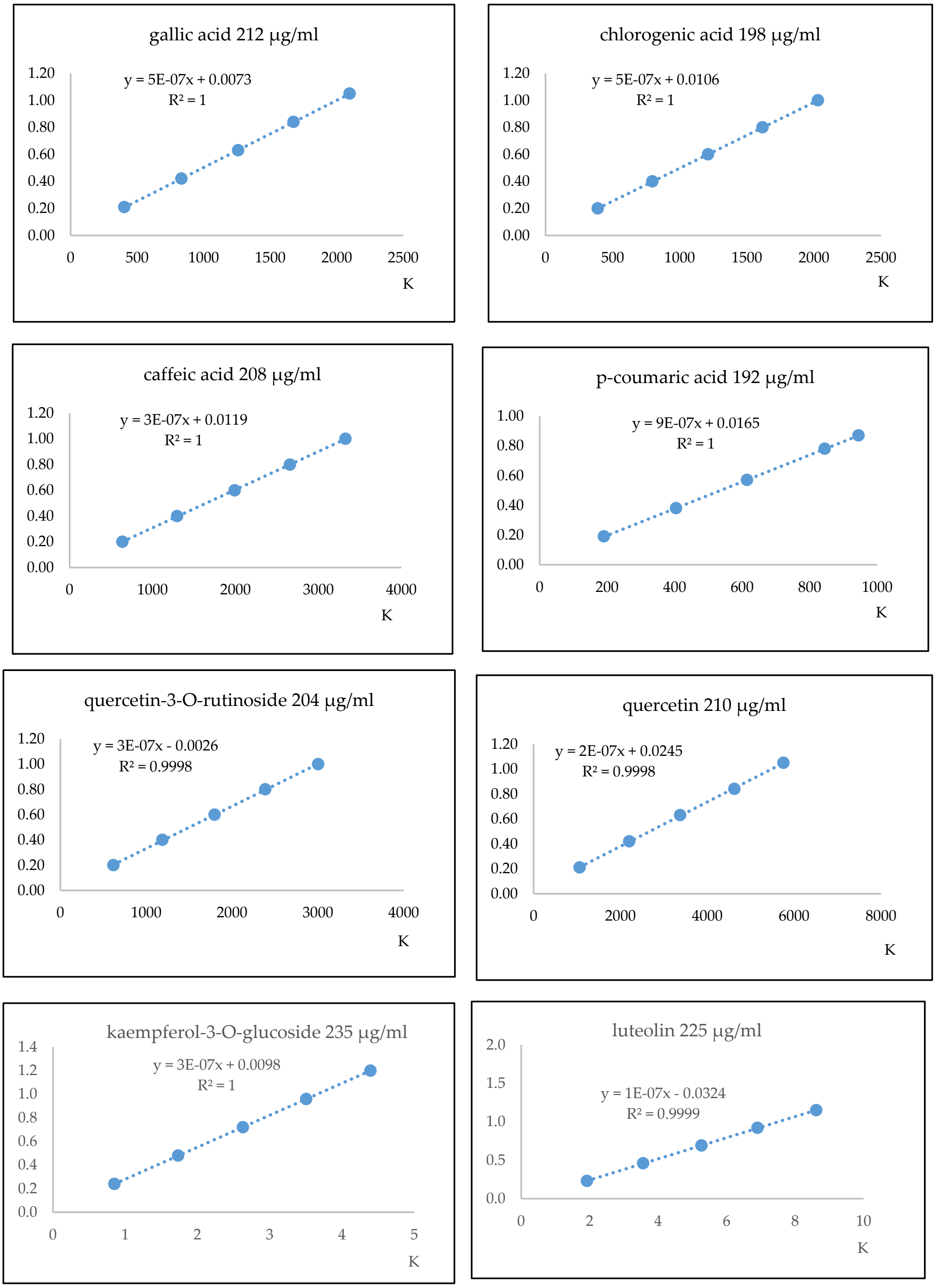

Figure S2. Standard curves for all compounds (polyphenols)identified in honeysuckle berries

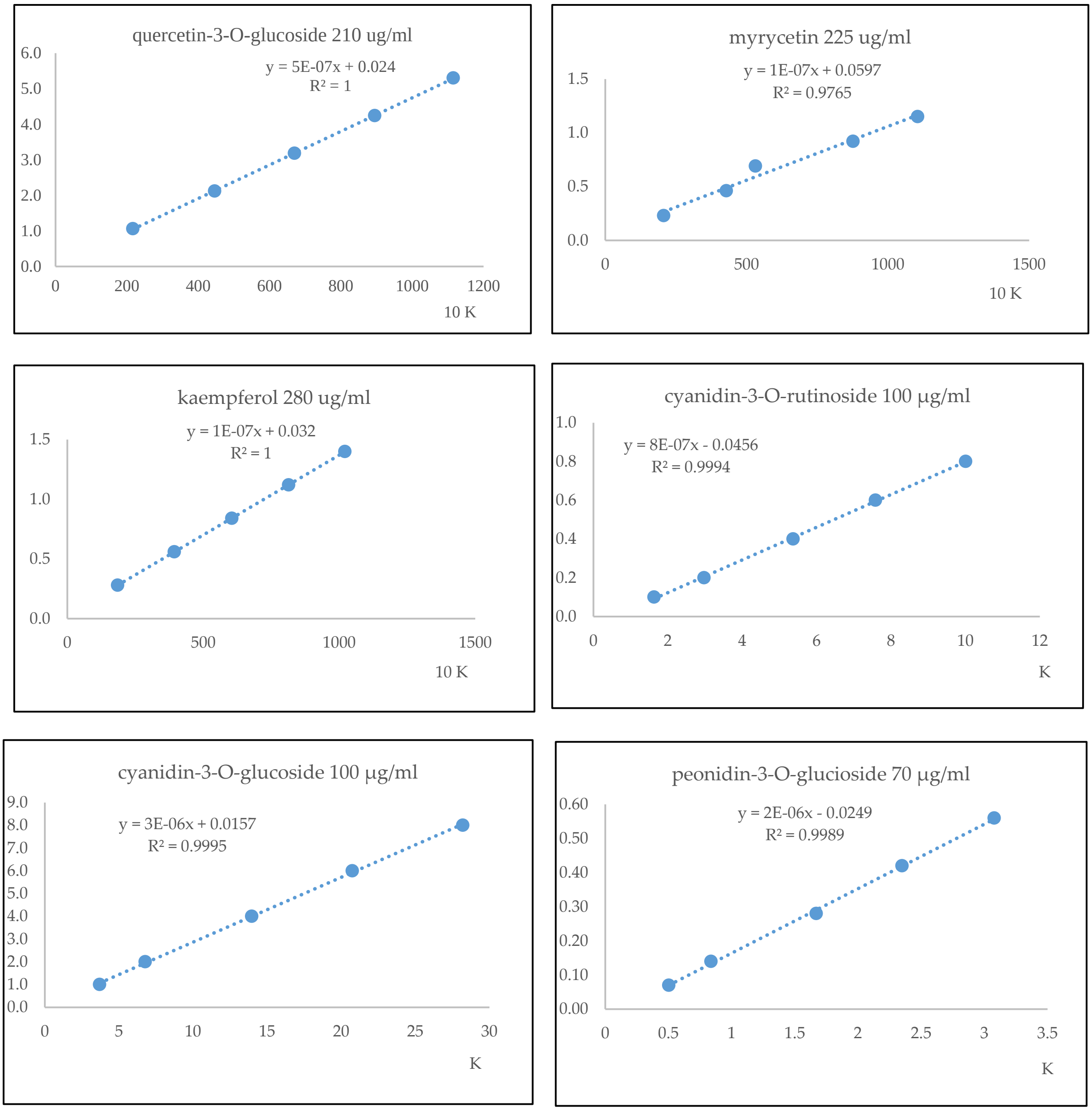

Figure S3. Standard curves for all compounds (polyphenols) identified in honeysuckle berries

Table S1. Correlation coefficients (R^2) among three analytical methods for antioxidant activity measurement

2018 organic	Antioxidant activity methods				2018 conventional	Antioxidant activity methods		
	ABTS	FRAP	DPPH			ABTS	FRAP	DPPH
ABTS					ABTS			
FRAP	0.912				FRAP	0.947		
DPPH	0.881	0.991			DPPH	0.850	0.992	
polyphenols	0.886	0.987	0.995		polyphenols	0.857	0.927	0.972
2019 organic	Antioxidant activity methods				2019 conventional	Antioxidant activity methods		
	ABTS	FRAP	DPPH			ABTS	FRAP	DPPH
ABTS					ABTS			
FRAP	0.877				FRAP	0.761		
DPPH	0.905	0.837			DPPH	0.617	0.933	
polyphenols	0.925	0.989	0.816		polyphenols	0.834	0.938	0.930
